# Direct assessment of foot kinematics during human gait using a dynamic cadaver simulator and a biplane X-ray fluoroscopy

**DOI:** 10.1186/1757-1146-7-S1-A105

**Published:** 2014-04-08

**Authors:** Kohta Ito, Naomichi Ogihara, Koh Hosoda, Masahiro Shimizu, Shinnosuke Kume, Takeo Nagura, Toshiyasu Nakamura, Nobuaki Imanishi, Sadakazu Aiso, Masahiro Jinzaki

**Affiliations:** 1 Department of Mechanical Engineering, Keio University, Yokohama 223-8522, Japan; 2 Department of Multimedia Engineering, Osaka University, Suita 565-0871 Japan; 3 School of Medicine, Keio University, Tokyo 160-8582 Japan; 4

## 

Direct measurement of detailed kinematics of individual anatomical structures in the foot during human locomotion is crucial for understanding morphofunctional roles of the human foot structure that mechanically interacts with the ground in a favourable manner to maintain stable gait. In the present study, we constructed a dynamic gait simulator to load and mobilize the cadaver foot and directly measured the three-dimensional kinematics of foot bones using a biplane X-ray fluoroscopy.

**Figure 1:**
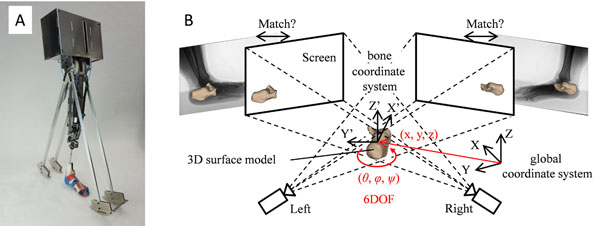
A robotic gait simulator to load and mobilize the cadaver foot in a manner similar to the way human foot moves and interacts with the ground during walking (A). Automatic method to register bone surface models with the two fluoroscopic images recorded by a biplanar dynamic X-ray fluoroscopic system (B).

A robotic gait simulator was developed to load and mobilize the cadaver foot in a manner similar to the way human foot actually moves and interacts with the ground during walking. The simulator has three legs, fore, middle and hind legs, with the cadaver foot fixed to the middle leg (Figure 1A). After the middle cadaver foot contacts the ground, the hind leg departs from the ground. The fore leg replicates the foot-contact with the ground in the next step and toe-off of the middle cadaver foot follows afterward. Tendons of tibialis anterior and soleus were connected to pneumatic actuators to apply forces at the appropriate moment to reproduce how the foot would function during walking.

A biplanar dynamic X-ray fluoroscopic system was developed with Shimadzu Corporation, Kyoto, Japan. The system consists of two sets of x-ray sources and flat panels with a resolution of 2688 x 2208 pixels. We recorded biplanar X-ray videos of foot movement using the system using the dynamic cadaver model at 15 fps. For direct measurement of 3D kinematics of the foot bones, we developed an automatic method to register bone surface models with the two fluoroscopic images (Figure 1B). Specifically, the 3D surface models of the foot were generated based on computed tomography (CT), and a similarity measure between occluding contours of the bone surface models with edge-enhanced fluoroscopic images was evaluated to reconstruct the position and orientation of each bone model in a 3D space. Collisions among the reconstructed bones were also evaluated to avoid penetration.

Using the biplanar X-ray fluoroscopic images and the proposed reconstruction methodology based on CT, we reconstructed 3D movements of the calcaneus, talus, navicular and cuboid when a human cadaveric foot walked on a flat surface using the simulator. The surface models of the four bones were successfully matched with the corresponding fluoroscopic images and the joint movements were quantified and visualized. The present methodology must be undergone further evaluation, but the proposed framework may serve as an effective tool for understanding the morphofunctional roles of the human foot structure during walking.

